# Kit foxes demonstrate adaptive compromise characteristics under intraguild predation pressure by coyotes in the Great Basin desert

**DOI:** 10.1038/s41598-024-61692-1

**Published:** 2024-06-24

**Authors:** Nadine A. Pershyn, Eric M. Gese, Erica F. Stuber, Bryan M. Kluever

**Affiliations:** 1 Department of Wildland Resources, Utah State University, Logan, UT 84322-5230 USA; 2 U.S. Department of Agriculture/APHIS/Wildlife Services, National Wildlife Research Center, Department of Wildland Resources, Utah State University, Logan, UT USA; 3 U.S. Geological Survey, Utah Cooperative Fish and Wildlife Research Unit Department of Wildland Resources and The Ecology Center, Utah State University, Logan, UT 84322 USA; 4 U.S. Department of Agriculture/APHIS/Wildlife Services, National Wildlife Research Center, Florida Field Station, 2820 E University Blvd, Gainesville, FL USA; 5 Present Address: Life Science II RM 251, 1125 Lincoln Dr, Carbondale, IL 62901 USA

**Keywords:** Behavioural ecology, Ecological modelling

## Abstract

Coyotes (*Canis latrans*) are believed to contribute to declining kit fox (*Vulpes macrotis*) numbers in the Great Basin desert through intraguild predation. Intraguild prey have been shown to exhibit adaptive compromise, whereby an animal increases selection for risky, but food-rich areas during times of food stress (i.e. winter). We evaluated the habitat selection of kit foxes in the Great Basin desert to elucidate if they demonstrated adaptive compromise as a method of coexisting with coyotes. We created 2nd order resource selection functions to analyze kit fox habitat selection associated with coyote relative probability of use (RPU), prey abundance, and type of soil substrate. In the summer, we found that kit fox selection for areas of relatively more abundant prey was not significant, and there was a small positive selection for coyote RPU. In the winter, we found a positive relationship between kit fox selection and prey abundance as well as a stronger selection for coyote RPU. These findings do follow the pattern of adaptive compromise. We also found kit foxes selected for silty and sandy soils, which are conducive to den construction, as they use dens seasonally for breeding but also year-round for multiple uses, including refugia from predators and extreme heat. Soil substrate appeared to be an important factor impacting kit fox habitat selection.

## Introduction

Following the reduction of large carnivores across North America, midsized carnivores such as coyotes (*Canis latrans*) expanded their distribution > 40% from their historic range of the eighteenth and nineteenth centuries^1^. This range expansion created novel interactions due to competition between midsized and small carnivores for habitat and prey^2,3^. Consequences of this expansion are often negative impacts on the subordinate carnivore species^4,5^. When the expanding, midsized carnivore has a generalist diet (e.g., coyotes), the effects can be coexistence or extirpation of the smaller, native carnivore^6^.

Kit foxes (*Vulpes macrotis*) are small, desert-adapted carnivores native to western North America’s arid rangelands^7^. Kit foxes have different conservation classifications throughout their range and are listed as vulnerable in the state of Utah^8^. Coyotes have expanded into arid ecosystems and now occur in most areas having abundant kit fox populations^5,9^. Intraguild predation^6^ by coyotes on kit foxes is the leading cause of kit fox mortality^3,5,10^. This example of intraguild predation is an extreme case of interference competition, as kit foxes are rarely consumed^2,3^. Researchers have proposed that increasing coyote populations drove a decline of kit fox numbers in the Great Basin desert, mainly via intraguild predation and competition^3,11,12^.

**Figure 1:**
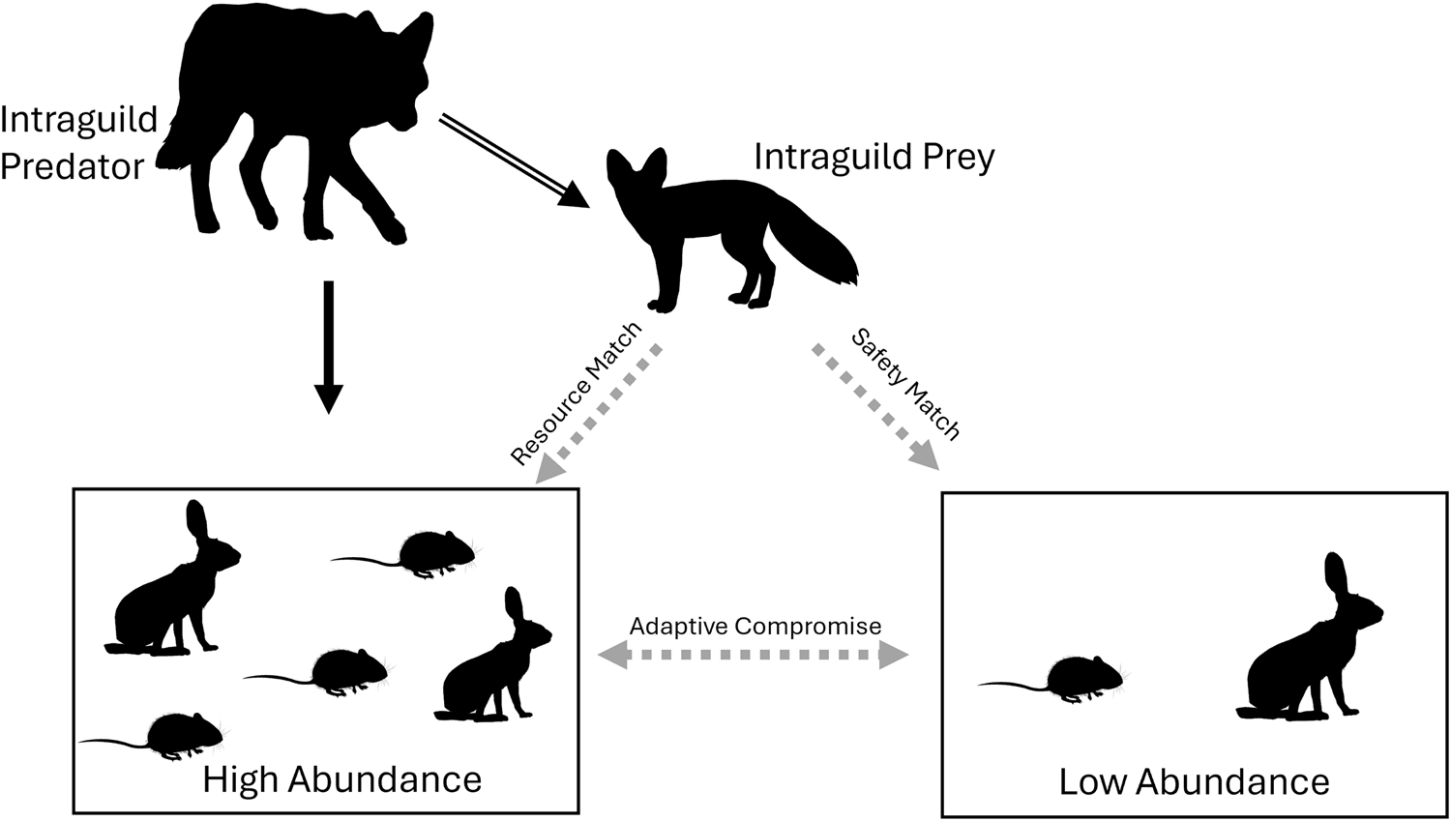
Matching strategies of intraguild prey. A resource match is the selection for high basal prey abundance, despite the increased risk of predation. A safety match is the selection for low prey abundance to prioritize safety over resource availability. Adaptive compromise is the shift between resource matching and safety matching based on environmental characteristics like seasonal prey availability.

Evidence of direct effects of intraguild predation, such as mortality, are readily documented; however, indirect effects, such as impacts on foraging behavior, space use, and habitat selection, are more difficult to quantify. A study of intraguild predation between coyotes and swift foxes (*Vulpes velox*) showed that coyote distribution matched the availability of the shared prey (resource match), while swift fox distribution inversely matched coyote distribution and therefore predation risk (safety match)^13^ (Fig. 1). However, it has also been shown that swift foxes will adjust their matching strategy dependent on prey availability; during the winter, when prey is scarce, they display a resource match of the shared basal prey, thereby trading security for resource availability during times of food stress (i.e., adaptive compromise;^14^; Fig. 1). Similarly, San Joaquin kit foxes (*V. m. mutica*), an endangered subspecies endemic to central California, displayed a safety match distribution by selecting habitats based on food availability and the risk of predation by coyotes and other predators^2,15^.

Kit foxes in the Great Basin desert have not been studied to evaluate their habitat selection relative to resource or safety matching. Dempsey et al.^16^ evaluated the spatial distribution of kit foxes in the Great Basin desert, but only assessed the impact of habitat covariates such as elevation, vegetation height, and soil type, and did not consider the presence of predators or prey. An in-depth study of how kit fox habitat selection relates to coyote space use and basal prey availability is critical to understanding how coyotes and kit foxes coexist in the Great Basin desert. Small mammals and leporids comprise 47% and 27% percent by volume of coyote diets and 64% and 8% of kit fox diets, respectively^17^. We evaluated small mammals and leporids as the shared basal prey for the two species due to the significant overlap. Our research objectives were to determine if kit foxes in the Great Basin desert displayed a safety match, a resource match, or if they exhibited adaptive compromise. A safety match would show kit foxes avoiding areas of higher predation risk and high prey abundance. A resource match would show kit foxes selecting for high prey abundance despite high coyote relative probability of use (RPU). We consider adaptive compromise to occur when an animal prioritizes safety from predation when food is abundant, but when food is scarce they switch to focus on resource availability regardless of risk. If kit foxes increase selection for coyote RPU and prey abundance in the winter, that would be indicative of adaptive compromise. We predicted that kit foxes in the Great Basin desert would exhibit adaptive compromise, based on the behavior of swift foxes, which utilized adaptive compromise as a matching strategy when faced with predation by coyotes^13^.

## Methods

### Study area

**Figure 2:**
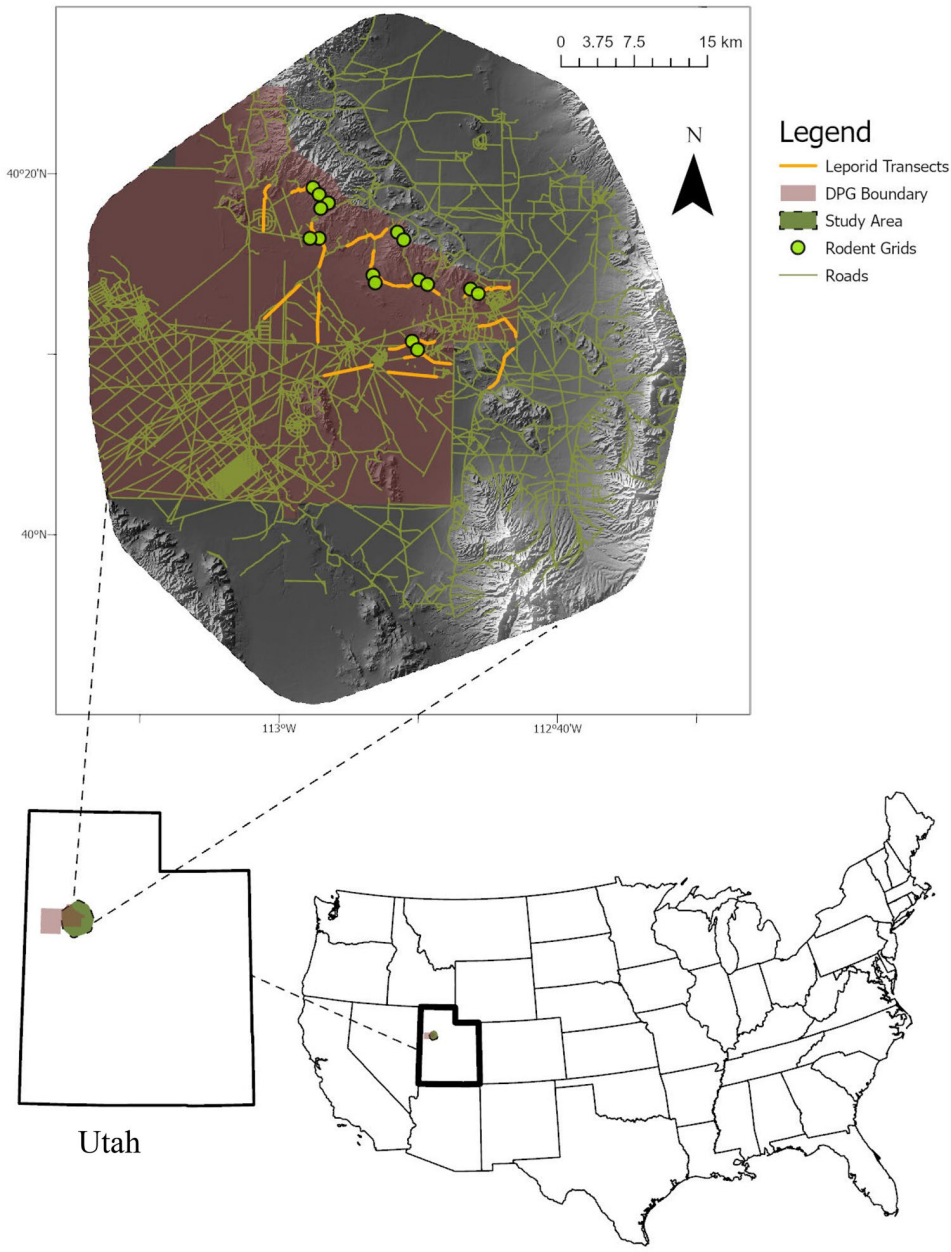
Rodent trapping grids (n = 16), and leporid spotlight transects (n = 15) in the study area. Dugway Proving Ground (DPG) and adjacent public lands, Utah, USA, 2010–2013. Background is a digital elevation model. Map created using Esri ArcGIS Pro 2.8.2 (https://www.esri.com/en-us/arcgis/products/arcgis-pro/overview).

We conducted this study in Tooele County, Utah, USA, within the eastern portion of the Dugway Proving Ground (DPG) and adjoining federal lands (Fig. 2). The DPG is a United States Army installation covering ~ 3000 km^2^ of Great Basin desert habitat. Urban development is < 1% of the study area^18^, and there are 215 and 3192 linear kilometers of paved and unpaved roads respectively. The DPG has been the site of ecological research since the 1950s, when Egoscue^19^ performed preliminary studies of kit foxes and found they were the most common carnivore on the DPG, a classification now held by coyotes^11^. Elevations ranged from 1298 to 3317 m. Annual mean air temperature was 12.7 °C (range: − 20.0–40.6 °C) and annual mean precipitation was 21.0 cm (range: 14.7–29.4 cm; U.S. Army Dugway Proving Ground, Meteorological Division). The Palmer Drought Severity Index (PDSI) during our study ranged from − 3.0 to 4.3 (mean = − 0.3, SD = 2.5) in the summer, and − 2.4–2.6 (mean = − 0.2, SD = 1.4) in the winter. The study site was in the Great Basin and was characterized as cold desert^12^. Winters were cold, summers were hot and dry, with most precipitation occurring in the spring. The study area comprised the following proportions of vegetation types: 31.3% desert scrub, 21.1% grassland, 17.4% sagebrush, 14.8% forest, 10.9% barren, 4.2% shrubland, and 0.3% developed.

Coyotes occurred throughout the DPG, but distribution of kit foxes was limited^16,20^. Other resident predators included bobcats (*Lynx rufus*), red-tailed hawks (*Buteo jamaicensis*), golden eagles (*Aquila chrysaetos*), and great horned owls (*Bubo virginianus*)^21^, all of which were not in the same foraging guild as kit foxes. Rodents, especially Ord’s kangaroo rats (*Dipodomys ordii*), were the primary prey of kit foxes^3,17,22^. Leporids, a major prey item for coyotes^3,23^, included black-tailed jackrabbit (*Lepus californicus*) and cottontail (*Sylvilagus spp*.). The small mammal fauna included Ord’s kangaroo rats, chisel-toothed kangaroo rats (*D. microps*), Great Basin pocket mice (*Perognathus parvus*), little pocket mice (*P. longimembris*), long-tailed pocket mice (*Chaetodipus formosus*), deer mice (*Peromyscus maniculatus*), Piňon mice (*P. truei*), western harvest mice (*Reithrodontomys megalotis*), northern grasshopper mice (*Onychomys leucogaster*), white-tailed antelope squirrels (*Ammospermophilus leucurus*), Townsend’s ground squirrels (Urocitellus townsendii), desert woodrats (*Neotoma lepida*), montane voles (*Microtus montanus*), sagebrush voles (*Lemmiscus curtatus*), and desert shrews (*Notiosorex crawfordi*)^23^.

### Animal capture and handling

All capture and handling protocols were reviewed and approved by the Institutional Animal Care and Use Committees (IACUC) at the United States Department of Agriculture’s National Wildlife Research Center (QA-1734) and Utah State University (#1438). Permits to capture and handle animals were obtained from the Utah Division of Wildlife Resources (COR #4COLL8322). All capture and handling procedures were in accordance with guidelines endorsed by the American Society of Mammalogists^24^.

#### Kit Fox capture, handling, and VHF tracking

Between January 2010 and November 2013, we captured 86 kit foxes via road based transect trapping^25^ and at known den sites^25,26^ using box traps (25 × 25 × 80 cm; Model 107; Tomahawk Live Trap LLC, Hazelhurst, Wisconsin) baited with hot dogs. Of these, we classified 44 as adults and 42 as juveniles (< 1 year old) at time of capture. We distributed road-based trapping transects to provide maximum coverage of the area and allow for increased likelihood of capturing most of the kit foxes occupying the study area^25^. We deployed traps in the evening and checked them early in the morning each day. We conducted road-based trapping each year on each transect for at least 8 nights during the breeding (15 December–14 April) and dispersal seasons (15 August–14 December). Due to concerns of overheating and the demands of natal care on female foxes, we did not conduct road-based trapping during the pup-rearing season (15 April–14 August—Dempsey et al. 2013) but we did trap and capture juvenile kit foxes at natal dens between 15 July and 14 August each year.

We weighed, determined sex of, ear tagged, and fitted each kit fox with a 30- 50-g very high frequency (VHF) radio-collar (Model M1930; Advanced Telemetry Systems, Isanti, Minnesota) weighing < 5% of body mass. We located animals > 3 times per week using a portable receiver (Model R1000; Communications Specialists, Inc., Orange, California) and a handheld 3-element Yagi antenna. We triangulated an animal’s location using ≥ 3 compass bearings, each > 20° but < 160° apart, recorded within 20 min^3,12^. We then calculated locations using program Locate III (Pacer Computing, Tatamagouche, Nova Scotia, Canada). We temporally distributed telemetry sampling by collecting 2 nocturnal locations and 1 den (resting) location each week to reduce autocorrelation among locations^27^. We attempted to locate each kit fox > 3 times per week to obtain 30 locations for each kit fox for each biological season as the minimum number of locations needed to adequately describe the home range^27^.

#### Coyote capture and handling

Methods for coyote capture followed Kluever and Gese^28^. We captured coyotes via helicopter net-gunning^29,30^ or foothold traps (#3 Soft Catch, Oneida Victor Inc., Euclid, OH) affixed with a trap tranquilizer device^31^. We staggered captures throughout the study, mainly between January 2010 and December 2012. Processing of coyotes included taking blood samples, affixing ear tags, and recording weight, sex, and morphological measurements. We determined age by tooth wear, tooth eruption and body size^32^, and we fitted adults with a 200 g global positioning system (GPS) radio-collar (Model M2220; Advanced Telemetry Systems, Isanti, MN). GPS collars ascertained a location every four hours. We captured coyotes throughout the study area and efforts were made to radio-collar only one individual per social group. We limited capture efforts to October through February of each year to not interfere with parturition and pup rearing.

#### Small mammal trapping

We used stratified random sampling to establish 16 total 50 × 50 m trapping grids in relation to permanent water sources^33^ (Fig. 2). Dispersal capabilities of our target species appeared to be less than the minimum distance between trapping grids with 428 m reported as the maximum dispersal distance for chisel-toothed kangaroo rats^34^ and 270 m exceeding the natural dispersal capabilities of Ord’s kangaroo rat^35^. The maximum dispersal distance for other species of kangaroo rats did not exceed 500 m^36^. During our study, no individual rodents were captured at multiple trapping grids. As such, we feel confident that dispersal capabilities of species investigated did not bias our findings through double-counting of a single individual at multiple grids. Established trapping grids were sampled repeatedly over the course of the study; new grids were not established every trapping session or year.

We sampled rodents in grids using a 7 × 7 configuration (49 traps [2 × 2.5 × 9′′; H.B. Sherman Traps, Inc., Tallahassee, Florida], 8.3 m spacing) for 4 consecutive nights (i.e., 4 capture occasions). We considered each 4-night sampling period as an individual trapping session. We conducted two trapping sessions on each grid per year: early session (1 May–30 June) and late session (1 August–30 September). We baited traps with a mixture of black sunflower and mixed bird seed. We identified all rodents captured to species, and they were ear tagged and measured (e.g., mass, tail length, hind foot length).

#### Leporid spotlight counts

We used nocturnal vehicle-based spotlight surveys^37^ to estimate relative abundance of leporids (black-tailed jackrabbits, cottontail rabbits) along fifteen 5-km road based transects (Fig. 2). While driving a vehicle along a transect at approximately 10–15 km/h, two observers scanned their respective side of the road and the road itself with a 3-million candlepower spotlight^38^. Surveys were conducted under clear and calm conditions between 1 h after dusk and 1 h before sunrise for three consecutive nights, resulting in a total of 45 separate spotlight counts per survey (i.e., three counts for each transect). Spotlight counts associated with each transect were then pooled across the three survey days. The order of transects surveyed each night was randomized. Once an animal was sighted the driver stopped the vehicle and the species of leporid was identified. We recorded the species, location, distance, and bearing to the animal for each sighting. We conducted surveys along the eight 5-km transects previously described. Surveys were temporally spaced so that we conducted one survey within each 4-month season based on energetic needs of coyotes: breeding (15 December–14 April), pup-rearing (15 April–14 August) and dispersal (15 August–14 December)^39^. Seasonal surveys were randomly selected across the 4-month period. When possible, we performed additional intra-season surveys, with ≥ 2 month spacing between surveys, during the pre- (2 extra surveys) and post-period (one extra survey). Spotlight surveys took place between September 2010 and August 2013.

### Data analysis

#### Small mammal abundance

Each of the 16 small mammal trapping grids was classified as one of the following vegetation types: barren ground (1 grid), desert scrub (5 grids), forest (2 grids), grassland (3 grids), sagebrush (3 grids), or shrubland (2 grids; landfire.gov, 2012). We classed the trapping sessions as summer (early session, May–June) and winter (late session, August–September). We calculated the number of individuals captured per grid in each vegetation type for both seasons of all study years (2010–2013). We then classified vegetation types as having low (1), medium (2), high (3), or very high (4) rodent abundance for each season of all study years (2010–2013) based on the number of individuals trapped per grid. We used quartile values across the study period as the distinction between each abundance score (i.e., 1st quartile and below was considered ‘low,’ etc.). We reclassified the LANDFIRE 2012 vegetation raster to represent the level of rodent abundance across the study area for desert scrub, forest, grassland, shrubland, sagebrush, barren, and developed. Vegetation types that could not be classified as one of those seven were represented as NA in the small mammal abundance raster and accounted for 0.35% of the study area. We combined small mammal and leporid abundance scores to create a single prey abundance layer and weighted small mammals as 2/3 of our prey raster, due to their biomass and prevalence in kit fox diets.

#### Leporid abundance

We classified all leporid locations from the spotlight surveys by the LANDFIRE 2012 vegetation class that they appeared in. We standardized the number of leporid sightings in each vegetation class based on transect, season, and the percentage of each vegetation class available within 400 m of the transects, as that was the maximum distance a leporid was spotted during the study. For example, if the number of leporids spotted in different vegetation types on a transect in one season was 40 in desert scrub, 7 in grassland, and 5 in sagebrush and the available vegetation surrounding the transect was 50% desert scrub, 30% grassland, and 10% sagebrush we calculated the ‘# of sightings’/‘% available’ to be 0.8 desert scrub, 0.23 grassland, and 0.5 sagebrush. Forest was not represented due to the location of survey transects. We then classified vegetation types as having low (1), medium (2), high (3), or very high (4) leporid abundance for each season of all study years (2010–2013) based on the standardized number of individuals spotted per vegetation type. We used quartile values across the study period as the distinction between each abundance score (i.e., 1st quartile and below was considered ‘low,’ etc.). We reclassified the LANDFIRE 2012 vegetation raster to represent the level of leporid abundance across the study area for desert scrub, grassland, shrubland, sagebrush, barren, and developed. Vegetation types that could not be classified as one of those six were represented as NA in the leporid abundance raster and accounted for 12.5% of the study area. We combined leporid and small mammal abundance scores to create a single prey abundance layer and weighted leporids as 1/3 of our prey raster due to their biomass and prevalence in kit fox diets.

#### Coyote relative probability of use

Using coyote GPS data, we developed a resource selection function (RSF) to model relative probability of use (RPU) by coyotes across our study area. RSFs use generalized linear models (GLMs) to evaluate which habitat and environmental covariates serve as predictors of wildlife space use. An RSF can be defined by a function where the relative probability of use, *w*(*x*), is related to a vector of *n* landscape covariates, *x*, and beta-coefficients, *β*, and is written in the log-linear form:

$${\text{w}}\left( {\text{x}} \right) = {\text{exp}}\left( {\beta_{{1}} {\text{x}}_{{1}} + \, \beta_{{2}} {\text{x}}_{{2}} + \cdots + \, \beta_{{\text{n}}} {\text{x}}_{{\text{n}}} } \right)$$

^43^

We used the **amt** package in R to run the RSF with 10 available locations being randomly generated for each observed location^40^. The RSF was calculated at the 2nd order of habitat selection, which is the placement of individual home ranges within the population range^41^. We created seasonal RSFs based on prey abundance, elevation, vegetation, distance to the closest road, and distance to the closest water source. We defined winter as October through March, and summer as April through September. We log transformed covariates that were ‘distance to’ features, and elevation was centered and scaled with a standard deviation of 1. Prey abundance was derived as described above. Vegetation was a categorical covariate derived from the LANDFIRE 2012 dataset with the following categories classified by plant physiognomy: agriculture, barren, desert scrub, developed, forest, grassland, other, riparian, sagebrush, and shrubland.

The area under the receiver operating curve (AUC), was used as a measure of model performance and was evaluated using out-ofs-ample data in a fivefold cross-validation procedure^42^. We followed the general classifications of ranking model accuracy in AUC scores; 0.5–0.7: low performance, 0.7–0.9: useful application, and > 0.9: high performance^43^. The coyote RSF was predicted across the landscape of the kit fox study area to predict coyote relative probability of use (RPU). The coyote and kit fox study areas encompassed 5,572 km^2^ and 3,231 km^2^ respectively, with 80% of the kit fox study area and the vast majority of kit fox location occurring within the coyote study area.

#### Kit fox resource selection function

Using the kit fox VHF locational data, we developed seasonal RSFs to analyze kit fox habitat selection using the **amt** package in R^40^. We defined winter as October through March, and summer as April through September. The RSF was calculated at the 2nd order of habitat selection, which is the placement of individual home ranges within the population range^41^. In the R package **amt**, we created a population range for kit foxes on the DPG using a 99% kernel density estimate (KDE) of all kit fox VHF locations combined^40^; 10 available locations per each used location were drawn from the population range. Habitat covariate values were extracted for both used locations and their paired available locations at the relevant season/year to account for covariates that varied throughout the study (i.e. prey abundance) and the analysis pooled data across years. We evaluated three models: a base model, a base + soil model, and a full model. Our base model contained the focal predation risk and prey density variables: coyote RPU and expected prey abundance. The full model included base covariates as well as type of soil substrate and distance to the closest road. We included soil substrate in several of our models, as kit foxes are a fossorial species that utilize a multitude of dens year-round round as refugia from predators and for their thermoregulation benefits, in addition to a place to raise their young^18,44^. Soil substrate is directly related to the ability to excavate dens. Other studies have shown that soil type can impact kit fox habitat selection^16,45^, so we found it prudent to include this variable. Soil substrate types were classified into the following categories: silt, fine sand, blocky loam, and gravel^16^.

Small mammals and leporids vary in biomass and composition of diet for coyotes and kit foxes^3,17,22^. Leporids comprise a larger portion of coyote diets than kit fox diets at only 8% by volume for kit foxes, while small mammals account for 64% of kit fox diet by volume^17^. We expect the magnitude of their impact on kit fox habitat selection to also vary, which is why we weighted their contributions to the prey abundance layer. Distance to roads was log-transformed. We checked for correlations between all covariates and any with a correlation higher than 0.7 were excluded from the models to avoid issues of collinearity. We used Akaike’s Information Criterion with a correction for small sample sizes (AIC_*c*_)^46^ to determine the best supported model, indicated by a ΔAIC of > 2. We validated model fit based on inspection of residuals and used semi-variograms to assess potential residual spatial autocorrelation.

## Results

### Small mammals

Between May 2010 and September 2013, we conducted 8 trapping sessions (2 sessions per year) for a total of 128 sampling occasions. We accumulated 25,088 trap nights, 5086 captures, and captured 2142 individual rodents. Abundance averaged 18.6 rodents (standard deviation (SD) = 15.61) per grid/session and ranged from 0 to 59 rodents/grid/session. Species and number of captures per species can be found in Supplemental Table 1. Each vegetation classification received a seasonal ranking based on the average individuals per grid in each year of the study, with forest having the highest abundance (Table 1).

**Table 1:** Small mammal and leporid abundance ranking for each vegetation classification, standardized respectively by average captures per grid and the percentage of each vegetation type available along each transect.

Prey category	Vegetation	2010 Summer	2010 Winter	2011 Summer	2011 Winter	2012 Summer	2012 Winter	2013 Summer
Small mammal	Barren	Medium	High	High	Medium	Medium	High	Low
	Desert Scrub	High	High	High	Medium	Very High	Very High	Low
	Forest	Very High	Very High	Very High	Very High	Very High	Very High	Medium
	Grassland	Medium	High	Medium	Low	Medium	High	Low
	Sagebrush	Low	Low	Low	Low	Medium	Medium	Low
	Shrubland	Low	Low	Low	Low	Low	Medium	Low
Leporid	Barren	Medium	Low	Low	Medium	High	High	Medium
	Desert Scrub	High	High	Medium	High	Very High	High	High
	Developed	Medium	Medium	Low	Low	Low	Low	Low
	Grassland	Medium	Medium	Medium	Medium	Very High	Low	Medium
	Sagebrush	High	Medium	Medium	Medium	Very High	High	High
	Shrubland	Medium	Medium	Low	High	Medium	High	Medium

Based on small mammal captures and leporid spotlight counts on the Dugway Proving Ground, Utah, USA, 2010–2013. Classifications were ranked according to the following distribution: small mammal- Low < 9 ≤ Medium < 17 ≤ High < 25 ≤ Very High; leporid- Low ≤ 0.2 < Medium ≤ 0.5 < High ≤ 1.2 < Very High. Raw values can be found in Supplemental Table 2.

### Leporids

Between September 2010 and August 2013, we conducted 12 leporid spotlight surveys and counted a total of 943 leporids. Leporid relative abundance across all surveys averaged 3.07 leporids/transect/night (SD = 2.60) and ranged from 0 to 19 leporids/transect/night. We grouped leporids based on the vegetation class in which they were observed and standardized the number of sightings by the percentage of each vegetation classification available on each transect. Each vegetation classification received a seasonal ranking based on the standardized number of sightings in each year of the study, with desert scrub having the highest abundance (Table 1).

### Coyotes

We radio-collared 31 coyotes (13 females, 18 males) between January 2010 and December 2012 and recorded ~ 65,000 GPS locations between January 2010 and December 2013. Our seasonal RSFs found negative relationships between elevation and distance to the closest water source (Table 2). Distance to roads was positively associated with coyote selection (Table 2). The area under the receiver operating curve (AUC) was 0.81 (SD = 0.0027) and 0.89 (SD = 0.0028) for the summer and winter RSFs, respectively, both of which show useful application and support the use of these models to predict coyote relative probability of use (RPU) across the landscape.

**Table 2:** Variables of the coyote (*Canis latrans*) resource selection function models for summer and winter.

Covariate	Summer			Winter		
	β estimate	Std. error	*p* value	β estimate	Std. error	*p* value
(Intercept)	− 2.99	5.89 e-2	< 0.0001	− 2.20	6.09 e-2	< 0.0001
Elevation	− 0.469	1.66 e-2	< 0.0001	− 0.854	2.16 e-2	< 0.0001
Vegetation: Agriculture	0.207	0.255	0.0.418	7.21 e-2	0.215	0.74
Vegetation: Desert Scrub	1.69	4.24 e-2	< 0.0001	0.742	4.03 e-2	< 0.0001
Vegetation: Developed	2.34	5.74 e-2	< 0.0001	1.16	6.58 e-2	< 0.0001
Vegetation: Forest	1.19	5.54 e-2	< 0.0001	0.900	5.60 e-2	< 0.0001
Vegetation: Grassland	1.18	4.39 e-2	< 0.0001	0.368	4.23 e-2	< 0.0001
Vegetation: Other	1.98	0.128	< 0.0001	1.21	0.154	< 0.0001
Vegetation: Riparian	1.88	0.295	< 0.0001	0.805	0.555	0.15
Vegetation: Sagebrush	1.63	4.59 e-2	< 0.0001	0.803	4.53 e-2	< 0.0001
Vegetation: Shrubland	2.11	4.59 e-2	< 0.0001	1.22	4.58 e-2	< 0.0001
Distance to road	0.283	9.99 e-3	< 0.0001	0.311	1.19 e-2	< 0.0001
Distance to water	− 2.96 e-4	2.31 e-6	< 0.0001	− 4.25e-4	3.67 e-6	< 0.0001
Prey abundance	4.23 e-3	4.25 e-3	0.32	3.25 e-2	6.13 e-3	< 0.0001

The model ‘Intercept’ represents the ‘Barren’ vegetation class, and coefficient estimates of other vegetation classes represent differences from the Intercept. Dugway Proving Ground, Utah, 2010–2013.

### Kit foxes

We captured 86 kit foxes (42 females, 44 males) and recorded ~ 7200 VHF locations between January 2010 and September 2013. Of those, only 42 foxes (23 females, 19 males) had ≥ 30 VHF points per season in at least one season and were considered for further analysis (~ 6000 VHF locations). Kit foxes included in the analysis had an average of 141 VHF locations (range: 30–543 locations, SD = 125) throughout the duration of the study. The summer and winter RSFs were created with 40 (23 females, 17 males) and 27 (15 females, 12 males) individuals (~ 3700 and ~ 2200 VHF locations), respectively, with 25 (14 females, 11 males) individual kit foxes contributing to both summer and winter analyses. Our top model based on AICc values was the full model which included coyote RPU, small mammal abundance, leporid abundance, soil, and distance to roads, with a ΔAIC_*c*_ for summer and winter of 0.58 and 45.6 to the next closest model, the soil model. In the summer the full and soil model were parsimonious. We include the full model in our results for easier comparison between seasons with the caveat that including distance to roads in the summer is not necessary.

**Figure 3:**
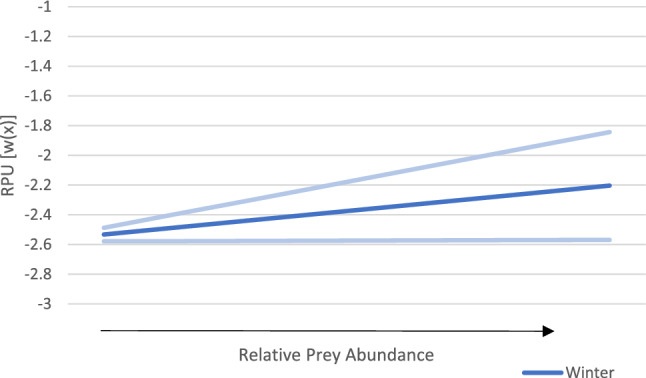
Effect of prey abundance on kit fox (*Vulpes macrotis*) relative probability of use (RPU) in the winter (blue) only, as prey abundance was not statistically significant during the summer. Upper and lower 95% confidence intervals (CI) shown in pale blue. As expected prey abundance increases, the probability of selection by kit foxes increases. Dugway Proving Ground (DPG) and adjacent public lands, Utah, USA, 2010–2013.

**Figure 4:**
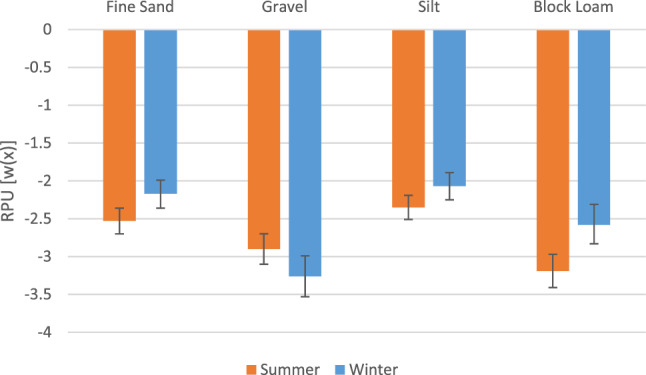
Effect of different soil substrate types on kit fox (*Vulpes macrotis*) relative probability of use (RPU) in the summer (orange) and winter (blue) with 95% confidence intervals (CI) represented by error bars. Dugway Proving Ground (DPG) and adjacent public lands, Utah, USA, 2010–2013.

**Figure 5:**
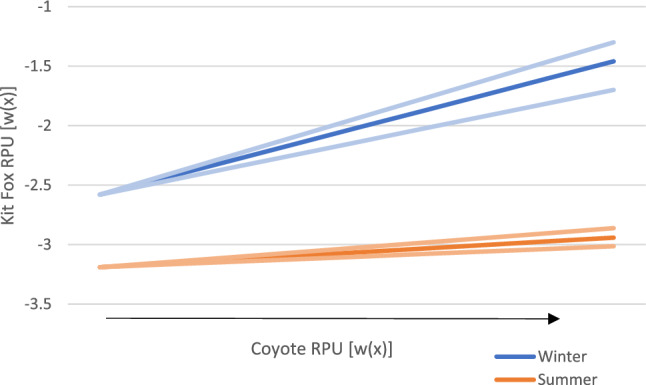
Effect of coyote (*Canis latrans*) relative probability of use (RPU) on kit fox (*Vulpes macrotis*) RPU in the summer (orange) and winter (blue) with 95% confidence intervals represented by pale orange and blue lines respectively Dugway Proving Ground (DPG) and adjacent public lands, Utah, USA, 2010–2013.

Our winter RSF showed a positive relationship between kit fox habitat selection and coyote RPU (β_coy_RPU_ = 0.14; Table 3, Fig. 5). Kit fox selection also increased with prey abundance (β_prey_ = 0.047; Table 3, Fig. 3). Blocky loam was the reference category for soil, and kit foxes selected for silt (β_silt_ = 0.51) and fine sand (β_fine sand_ = 0.41), while the least selected soil type was gravel (β_gravel_ = − 0.68; Table 3, Fig. 4). Kit foxes had a negative relationship with distance to roads (β_roads_ = − 0.2; Table 3).

**Table 3:** Variables from the top resource selection function models for kit foxes (*Vulpes macrotis*) in summer and winter.

Covariate	Summer			Winter		
	β estimate	Lower CI	Upper CI	β estimate	Lower CI	Upper CI
(Intercept)	− 3.19	− 3.41	− 2.97	− 2.58	− 2.85	− 2.33
Soil: Fine Sand	0.66	0.49	0.83	0.41	0.23	0.60
Soil: Gravel	0.29	0.089	0.49	− 0.68	− 0.95	− 0.41
Soil: Silt	0.84	0.68	1.00	0.51	0.33	0.69
Distance to Road	0.037^a^	− 0.01	0.085	− 0.20	− 0.25	− 0.14
Coyote RPU	0.031	0.022	0.041	0.14	0.11	0.16
Prey Abundance	0.001^a^	− 0.029	0.031	0.047	0.0013	0.092

Blocky loam was the reference (Intercept) category for soil. RPU is the relative probability of use.

^a^Variables where estimated 95% confidence interval (CI) overlaps 0. Dugway Proving Ground, Utah, 2010–2013.

Our summer RSF also indicated a positive correlation with coyote RPU (β_coy_RPU_ = 0.031; Table 3, Fig. 5), though selection for prey abundance was not statistically significant (Fig. 3). Blocky loam was the reference category for soil, and kit foxes selected most strongly for silt (β_silt_ = 0.84) and fine sand (β_fine sand_ = 0.66; Table 3, Fig. 4), then for gravel (β_gravel_ = 0.29; Table 3, Fig. 4). Kit foxes had a positive relationship with distance to roads (β_roads_ = 0.037; Table 3); however, the soil model (which excluded distance to roads) was parsimonious to the full model, indicating the unimportance of distance to roads.

## Discussion

The output from our kit fox resource selection functions (RSFs) indicated coyote RPU positively influenced kit fox habitat selection in both seasons, which we did not anticipate (Fig. 5). Higher relative probability of use by coyotes increases the risk of predation, so we anticipated kit foxes would avoid those areas in the summer (safety match) and utilize them in the winter (resource match). A multitude of studies have found evidence that kit foxes, and foxes in general, usually avoid areas with high coyote abundance^2,13,15^. Our results were similar to the findings of a study performed within the same general study area^45^ that reported kit fox detection was higher in areas with greater coyote activity, though employing an occupancy framework. Another study^44^ also found that kit foxes did not avoid coyotes and posited that the two species can coexist if kit foxes exploited certain prey species better than coyotes. Other studies have found that in some cases swift foxes^47^ and gray foxes^48^ also do not spatially avoid coyotes.

We observed a non-significant or low selection for prey abundance, suggesting that prey availability is not a top driving factor of kit fox habitat selection. Other studies have found that kit fox use of habitats with the highest values for diversity and abundance of prey was lower than anticipated on the DPG^20^. Selection for prey was non-significant in the summer, a time when prey is more plentiful on the landscape. However, in the winter we did find selection for prey abundance. While we did not observe a shift from a safety match to a resource match, we did see an increase in the selection for both coyote RPU and prey abundance in the winter, which could be indicative of adaptive compromise. The shift of prey availability not influencing habitat selection during the summer to having an effect in the winter suggests that habitat selection based on prey availability varies seasonally and is most important in the winter, a time when food is scarce. Furthermore, the stronger effect of coyote RPU on kit fox habitat selection seen in winter demonstrates that the selection for prey availability is likely accompanied by higher predation risk. We did not find any avoidance of coyotes by kit foxes in either season, which could be due to coyotes’ widespread nature making it impossible for kit foxes to avoid them completely^45^.

Lonsinger et al.^45^ posited that coexistence between kit foxes and coyotes on the DPG was facilitated by kit foxes employing a combination of broad-scale safety matching and fine-scale resource matching. Our 2nd order RSF would likely only have reflected broad scale habitat selection, and we did not observe a safety match. Schooley et al.^49^ found differing diel activity patterns for kit foxes and coyotes and suggested kit foxes might use temporal rather than spatial avoidance. Additionally, there is some evidence that time of day may impact the shift between kit foxes focusing on nutrient intake (typically at night) or predator vigilance (typically during the day;^3^). A future study might use more frequent GPS fixes to analyze fine-scale habitat selection of kit foxes and consider including interaction terms for time of day. Fine scale temporal factors, such as moon phase, could also be explored, especially as the use of GPS transmitters for small mammalian carnivores like kit foxes becomes more feasible.

Soil types that are conducive to denning may be the most important factor of kit fox habitat selection. We found a strong selection by kit foxes for silt and fine sand as the soil substrate (Fig. 4), similar to other studies showing kit fox preference for these types of soils^16,45^. Kit foxes utilize numerous dens year-round and outside of hunting forays, which are typically nocturnal, they are always associated with dens^18,44^. This would explain the observed selection for locations where the soil substrate was conducive to excavating dens^50^. Kit fox dens can contain multiple entrances, allowing for quick access when faced with a threat^18^. Dens provide refuge from coyotes, which may be impossible to spatially avoid due to their ubiquitous presence across the landscape^45^. By remaining near a den, it offers a safe retreat in the event of an encounter with a coyote. Soil type is an important factor in habitat selection for other denning carnivores; the soil of Arctic fox (*Alopex lagopus*) dens was typically sandy loam to sand^51^ and European badgers (*Meles meles*) preferred easily dug, well-drained soils when establishing diurnal resting dens^52^.

We saw inconsistent selection for distance to roads for kit foxes. In the summer kit foxes avoided roads, while in the winter they selected for roads (Table 3). Coyotes selected for roads in both seasons (Table 2). Carnivores have been shown to have varying relationships with roads temporally^53^, seasonally^54^, and even within seasons^55^. Investigating selection for/against roads is important as many survey methods, including some implemented here, used road-based methodologies, which can introduce bias if there is a strong relationship between carnivores and roads. Here though, selection for/against roads by kit foxes and coyotes was inconsistent and relatively minor—especially in the summer when the model that included it was parsimonious with one that excluded it, which justifies our use of roads for surveys to collect largely unbiased data throughout the study.

Studies of carnivores found that top-down pressures on intraguild prey are stronger than bottom-up, which is typical of a safety match distribution. Goodheart et al.^56^ found that predictors of prey density consistently had weaker effects on the movement of African wild dogs (*Lycaon pictus*) than the presence of lions (*Panthera leo*). Safety matching has been seen in swift foxes^13^, African wild dogs^57^, and San Joaquin kit foxes^2,15^. Alternatively, a study of sympatric felids found that prey availability best explained habitat-use and found no evidence of negative associations of larger felids on smaller felid occupancy^58^. There can be differences in carnivore guild interactions even between canids and felids, showing the diversity and complexity of intraguild interactions.

Our study focused on understanding the complex and dynamic relationship between coyotes and kit foxes in the Great Basin desert. When Egoscue^19^ studied kit foxes on the DPG over 60 years ago, they were the most abundant carnivore and had minimal pressures from intraguild predation by coyotes. Now, coyotes are present across the landscape, making it difficult for kit foxes to avoid them^45^. Our research, in combination with other studies, showed that kit foxes on the DPG are surviving alongside coyotes but the population has declined^3,16,45^. Kit fox densities decreased from 0.15 foxes/km^2^ in the mid-1950s^19^ to 0.02 foxes/km^2^ in 2014^22^. Concerns over the feasibility of kit foxes maintaining healthy population levels appears justified^8^. Efforts to reduce coyote abundance in the area to promote declining and sensitive kit fox populations must recognize the highly adaptive nature of coyotes. Coyotes appear to be desert adapted carnivores, not reliant on artificial water sources, so removal of those water sources is not a viable method to reduce coyote abundance^23,28,59^. If removal of coyotes from the landscape proves to be an untenable management solution, a strong understanding of the survival mechanisms of kit foxes will be necessary for wildlife managers to promote population persistence. This will ensure that management plans are targeting true drivers of kit fox survival and using resources efficiently and effectively. The intricacies of their predator avoidance and hunting/foraging strategies will be key components of sustaining future populations. For example, determining which vegetation types, and the underlying driving mechanisms (e.g., average vegetation height, shrub density), expose kit foxes to the highest risk of intraguild predation might allow for managers to focus conservation and management efforts in identified “high risk” areas.

### Supplementary Information

Supplementary Tables.

## Data Availability

The datasets analyzed during the current study are available from the corresponding author on reasonable request.
