# Supplementary material for: Kit foxes demonstrate adaptive compromise characteristics under intraguild predation pressure by coyotes in the Great Basin desert
Source: Sci Rep. 2024 Jun 24;14:14446. doi: 10.1038/s41598-024-61692-1 (PMC11194276; doi:10.1038/s41598-024-61692-1)
Supplement: Supplementary file 1 — Supplementary Tables. [file 41598_2024_61692_MOESM1_ESM.docx]

# KIT FOXES DEMONSTRATE CHARACTERISTICS OF ADAPTIVE COMPROMISE UNDER INTRAGUILD PREDATION PRESSURE BY COYOTES IN THE GREAT BASIN DESERT

**Authors:** Nadine A. Pershyn^a*^, Eric M. Gese^b^, Erica F. Stuber^c^, Bryan M. Kluever^d^

^a^ Department of Wildland Resources, Utah State University, Logan, UT 84322-5230, USA

^b^ U.S. Department of Agriculture/APHIS/ Wildlife Services, National Wildlife Research Center, Department of Wildland Resources, Utah State University, Logan, UT, USA

^c^ U.S. Geological Survey, Utah Cooperative Fish and Wildlife Research Unit Department of Wildland Resources & The Ecology Center Utah State University, Logan, UT 84322

^d^ U.S. Department of Agriculture/APHIS/ Wildlife Services, National Wildlife Research Center, Florida Field Station, 2820 E University Blvd, Gainesville, FL, USA

*Corresponding author: Nadine Pershyn (nadine.pershyn@siu.edu)
Present address:
1125 Lincoln Dr
Life Science II RM 251
Carbondale, IL, USA 62901

**Supplemental Table 1:** Summarized rodent capture results across all trapping grids and sessions at Dugway Proving Ground, Utah, 2010–2013. Total number of trap nights was 25,088 (Kluever et al., 2016).

| Species | Number of captures | Percentage of captures | Number of individuals | Percentage of individuals |
| --- | --- | --- | --- | --- |
| *Dipodomys ordii* | 3,507 | 68.95 | 1,423 | 66.34 |
| *Peromyscus maniculatus* | 798 | 15.69 | 374 | 17.44 |
| *Dipodomys microps* | 306 | 6.02 | 133 | 6.20 |
| *Chaetodipus formosus* | 171 | 3.36 | 62 | 2.89 |
| *Onychomys leucogaster* | 95 | 1.87 | 61 | 2.84 |
| *Reithrodontomys megalotis* | 81 | 1.59 | 49 | 2.28 |
| *Perognathus mollipilosus* | 40 | 0.79 | 16 | 0.75 |
| *Neotoma lepida* | 14 | 0.28 | 9 | 0.42 |
| *Peromyscus truei* | 12 | 0.24 | 6 | 0.28 |
| *Ammospermophilus leucurus* | 8 | 0.16 | 7 | 0.33 |
| *Lemmiscus curtatus* | 4 | 0.08 | 1 | 0.05 |
| *Perognathus longimembris* | 1 | 0.02 | 1 | 0.05 |

| **Prey Category** | **Vegetation** | **2010** | | | | **2011** | | | | **2012** | | | | **2013** | |
| --- | --- | --- | --- | --- | --- | --- | --- | --- | --- | --- | --- | --- | --- | --- | --- |
|  |  | **Summer** | | **Winter** | | **Summer** | | **Winter** | | **Summer** | | **Winter** | | **Summer** | |
|  |  | **Mean** | **SD** | **Mean** | **SD** | **Mean** | **SD** | **Mean** | **SD** | **Mean** | **SD** | **Mean** | **SD** | **Mean** | **SD** |
| **Small Mammal** | Barren | 12 | 0 | 21 | N/A | 23 | N/A | 11 | N/A | 11 | N/A | 23 | N/A | 9 | N/A |
|  | Desert Scrub | 17.2 | 7.4 | 24 | 10.6 | 22.2 | 17.2 | 16.4 | 13.7 | 27.4 | 12.5 | 28 | 11.8 | 4.6 | 4.1 |
|  | Forest | 26.5 | 8.9 | 25 | 0 | 30.5 | 0.7 | 25 | 7.1 | 42.5 | 0.7 | 34 | 2.8 | 12 | 1.4 |
|  | Grassland | 9.2 | 2.2 | 20 | 10.6 | 14 | 6.6 | 4.3 | 1.5 | 18.3 | 8.7 | 24 | 8.5 | 6.3 | 2.1 |
|  | Sagebrush | 10.5 | 5.9 | 8.7 | 1.2 | 9.3 | 4.9 | 6.7 | 1.5 | 18.7 | 5.5 | 16 | 4.6 | 6 | 3.6 |
|  | Shrubland | 7.8 | 5.9 | 5 | 2.8 | 1.5 | 0.7 | 0.5 | 0.7 | 4 | 2.8 | 9.5 | 6.4 | 5.5 | 6.4 |
| **Leporid** | Barren | 0.5 | 0.71 | 0.00 | 0.00 | 0.00 | N/A | 0.50 | 0.71 | 1.14 | N/A | 0.89 |  | 0.50 | N/A |
|  | Desert Scrub | 1.14 | 0.39 | 0.87 | 0.38 | 0.38 | N/A | 1.11 | 0.19 | 1.68 | N/A | 0.97 | 0.09 | 0.78 | N/A |
|  | Developed | 0.50 | 0.71 | 0.33 | 0.58 | 0.00 | N/A | 0.00 | 0.00 | 0.00 | N/A | 0.00 | 0.00 | 0.00 | N/A |
|  | Grassland | 0.48 | 0.10 | 0.30 | 0.30 | 0.41 | N/A | 0.43 | 0.12 | 1.62 | N/A | 0.16 | 0.02 | 0.29 | N/A |
|  | Sagebrush | 0.73 | 0.13 | 0.48 | 0.06 | 0.36 | N/A | 0.50 | 0.13 | 1.45 | N/A | 0.64 | 0.13 | 0.57 | N/A |
|  | Shrubland | 0.25 | 0.12 | 0.44 | 0.19 | 0.17 | N/A | 0.67 | 0.24 | 0.50 | N/A | 1.08 | 0.82 | 0.35 | N/A |

**Supplemental Table 2**: Small mammal and leporid abundance ranking for each vegetation classification, standardized respectively by average captures per grid and the percentage of each vegetation type available along each transect. Based on small mammal captures and leporid spotlight counts on the Dugway Proving Ground, Utah, USA, 2010-2013.
